# Comparative analysis of racing performance following surgical correction of epiglottic entrapment in standardbreds and thoroughbreds

**DOI:** 10.3389/fvets.2024.1479144

**Published:** 2024-11-19

**Authors:** Alannah M. Norton, Sarah M. Rosanowski, Tom O’Brien

**Affiliations:** ^1^Stutsel Veterinary Services, Bathurst, NSW, Australia; ^2^Consultant, Palmerston North, New Zealand; ^3^Avenel Equine Hospital, Avenel, VIC, Australia

**Keywords:** equine, epiglottic entrapment, upper respiratory tract surgery, racehorse, standardbred, thoroughbred

## Abstract

**Introduction:**

Epiglottic entrapment (EE) is a common cause of poor performance in racing standardbreds (STBs) and thoroughbreds (TBs). There is limited information published on the condition in STB racehorses and limited information on factors associated with return to racing available for either breed.

**Methods:**

This study investigated and compared the pre surgical findings, post-surgical complications and racing performance in STBs and TBs undergoing surgery for correction of EE. Associations between signalment and pre-surgical findings, and the presence of complications and racing post-surgery were compared using logistic regression analyses. Racing performance following surgery was compared to maternal siblings using conditional logistic regression.

**Results:**

There were 82 cases where epiglottic entrapment surgery was performed at least once: 33 STB cases and 49 TB cases. In total, 74% of cases raced following surgery. Cases with severe inflammation post-surgery (*p* < 0.001), airway complications (*p* < 0.001) or an epiglottic re-entrapment (*p* < 0.01) were at a lower odds of racing post-surgery. TB cases were 5.5 times (95% Confidence interval 1.61 to 18.73) more likely to race than their maternal siblings (*p* < 0.01). TB cases took 79% longer to return to racing compared to STB cases (*p* < 0.001). Horses that had raced prior to surgery were 4.1 times more likely to race after surgery than those that had not (95% CI 1.01–16.67).

**Conclusion:**

Horses with post operative complications were at a lower odds of racing post surgery. In the current study, TBs with EE are more likely to race compared to their maternal half siblings however take longer to return to racing compared to STBs.

## 1 Introduction

Epiglottic entrapment (EE) is a common condition affecting the equine larynx with a reported incidence of 0.9–5% (1–3). The condition is diagnosed endoscopically and occurs when the sub epiglottic tissue (an extension of the aryepiglottic folds) folds dorsally and entraps the epiglottis. It is generally considered to be a performance inhibiting condition, however there are reports of EE occurring in clinically normal, racing horses (2, 3). In one report, the diagnosis of epiglottic entrapment was associated with an improved racing performance or higher stakes earnings (3). Despite these observations, it is widely accepted to be a cause for increased respiratory noise and poor performance and is therefore classed as a ‘fail’ for Thoroughbreds (TBs) and Standardbreds (STBs) undergoing post sale upper respiratory tract endoscopy in Australia.

Different surgical techniques have been described, including transection of the entrapping membrane via transendoscopic diode laser, transection via oral hook bistoury knife, transection via trans-nasal hook bistoury knife, resection of the entrapping tissue using an endoscopic snare and via laryngotomy incision (4–11).

Previous studies have evaluated racing performance and post-operative complications in TBs following surgical correction of EE (6–9, 11–15). Despite the condition being recognised in STB racehorses, there are few reports in racing outcome of this breed. There are also no reports across a mixed population of racehorses (yearlings, raced, unraced, performed horses) or STBs describing the outcome in clinical cases. There are inherent differences in the training and racing patterns between the two breeds and associated different respiratory and physiological requirements (16–19). This study aims to describe the racing performance and examines the variables contributing to a successful return to racing in both TB and STB racehorses undergoing surgical correction of EE at Agnes Banks Equine Clinic, Hawkesbury, NSW Australia (ABEC) between 2011 and 2022 using logistic regression analysis. The aim will be addressed in these three objectives: (1) To describe the clinical presentation, post-surgical complications and prognosis for TB and/or STB horses following EE surgery, (2) to describe post-surgical racing performance of TB and STB horses following EE surgery and (3) to compare racing performance between horses undergoing EE surgery and their maternal siblings.

## 2 Methods

### 2.1 Retrospective study design and population

A retrospective cohort study of veterinary clinical records for TB and STB racehorses registered with their respective studbook (i.e., had the potential to be racehorses) presenting to ABEC for surgical treatment of EE between January 2011 to July 2022.

### 2.2 Case definition and identification

Cases were identified through a text search of clinical notes for “epiglottic entrapment,” “EE,” “epiglottis,” and “entrapment” between the study dates. TB or STB cases that underwent surgical correction of epiglottic entrapment via oral hook transection of the entrapping membrane under general anaesthesia were identified. Horses were excluded if they were not registered with the Australian Stud Book (ASB) or Harness Racing Australia (HRA), had already been retired from racing or used for a discipline other than racing (e.g., showjumping or eventing) at the time of admission, or any other technique of correction was used.

### 2.3 Data collection and variable management

Information retrieved from veterinary clinical records included age at admission, sex, presenting clinical signs, any medical pre-treatment of the epiglottic entrapment, pre surgical endoscopy findings, surgery date, post-surgical endoscopic findings, post-surgical management, presence of longer-term upper airway pathology, duration of hospitalisation, and whether a repeat surgery was conducted. If records regarding longer term upper airway pathology were missing, owners or trainers were called to obtain this information.

For consistency, all horses (TB and STB) were aged based on the TB southern hemisphere birthdate of the 1st of August. Horses were considered to be “young” if surgery occurred prior to the beginning of their 2-year-old year. Sex was described as geldings, fillies (including both mares and fillies) and colts (including both stallions and colts).

Pre-operative endoscopy findings were graded using a novel severity scoring system. Entrapping membrane thickness (whether oedema or fibrous) was graded as mild (=1), moderate (=2) or severe (=3) along with the presence of ulceration (ulcerated = 1, non-ulcerated =0). The scores were added together to calculate an overall severity score. This scoring system provided and objective framework to report on the overall inflammation of the entrapping membrane.

**Figure 1:**
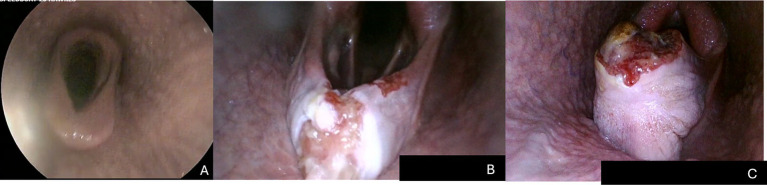
(A) Thin entrapping membrane (grade 1) with no ulceration (severity score grade 1). (B) Moderately thickened (grade 2) entrapping membrane with ulceration (severity score grade 3). (C) Markedly thickened (grade 3) entrapping membrane with ulceration (severity score grade 4).

For each horse, the number of career races, the number of races pre- and post-surgery, the total earnings ($AUD) and earning pre- and post-surgery were collated from the HRA^1^ website for STBs and from www.racing.com for TBs. Data collection ended in March 2023. A binary variable of raced pre-surgery (yes, no) and raced post-surgery (yes, no) was generated. The time between surgery and return to racing in days was then generated. For horses that were < 2 years of age at the time of surgery and underwent surgery late in the study period (after 2020), their racing performance was not considered, as it was unlikely to be representative (Figure 1).

### 2.4 Epiglottic entrapment surgery

The surgical technique was reviewed to confirm consistent technique across all cases. Horses were premedicated with flunixin meglumine (1.1 mg/kg IV), dexamethasone (0.1 mg/kg IV) and xylazine (1.1 mg/kg IV). General anaesthesia was induced with ketamine (2.5 mg/kg IV) and diazepam (0.2 mg/kg IV) through a jugular intravenous catheter. The horses were moved into theatre and placed in right lateral recumbency on an operating table. In all surgeries performed between 2021 and 2022 a nasal tube was placed immediately after induction of anaesthesia for supplementation with 100% oxygen at a rate of 5 L/h.

**Figure 2:**
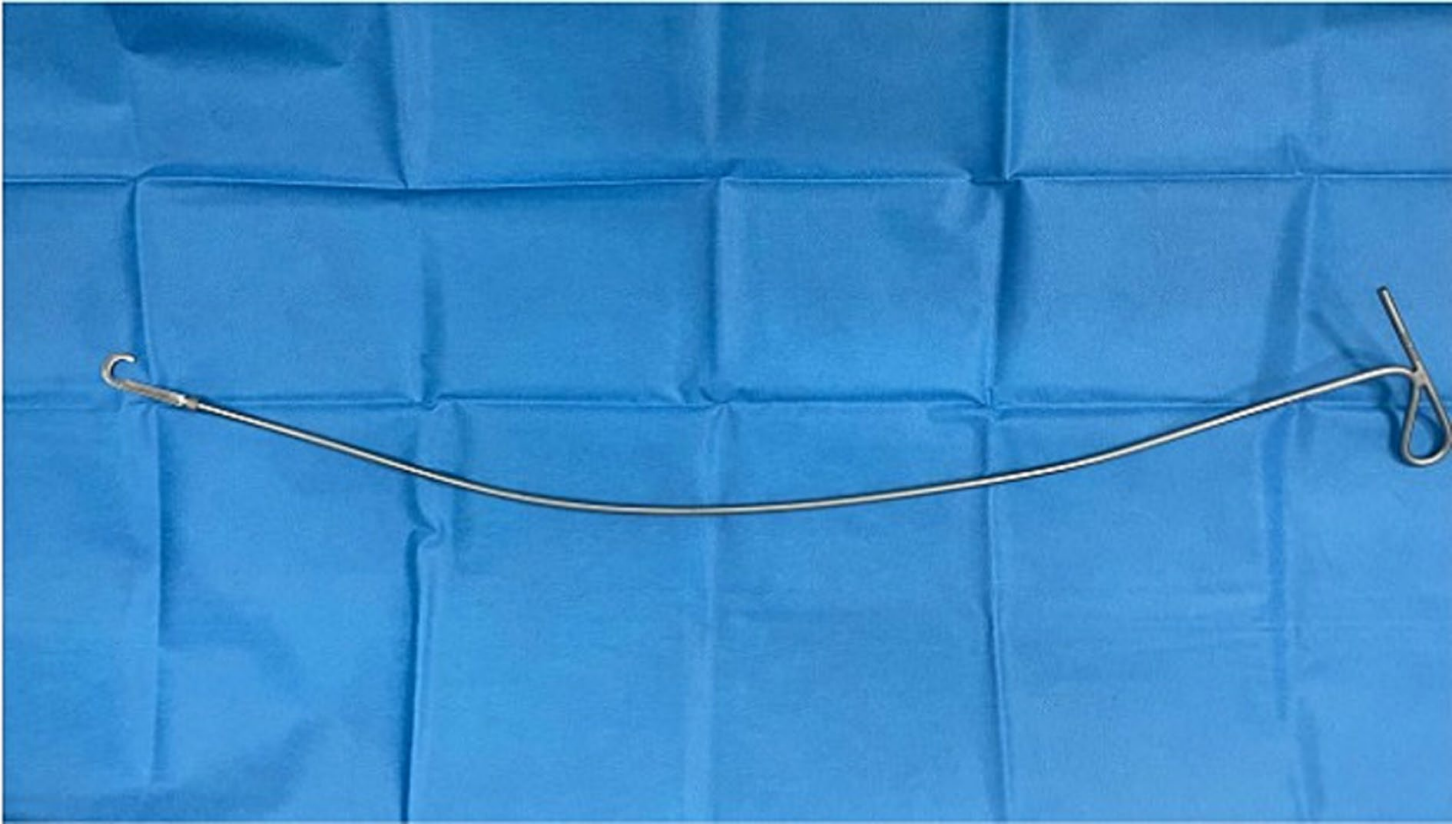
Sharp hook used to transect the entrapping membrane.

A Hausman’s mouth gag was placed and 50–100 mL lidocaine 2% applied topically to the larynx through the biopsy channel of a video endoscope placed orally. The entrapping membrane was palpated with the surgeon’s dominant hand and if necessary, the epiglottis manually re-entrapped. The epiglottis was pushed dorsally, and the sharp end of the hook placed in the centre of the most ventral part of the entrapping membrane (10, 14, 16) (Figure 2). After palpation to confirm the epiglottis was clearly not incorporated into the hook, the surgeon used the non-dominant hand to place manual traction on the end of the hook and cut the membrane whilst the hand in the pharynx was used to shield and control the sharp blade. Complete resection of the entrapping membrane was confirmed via intraoperative oral video endoscopy and palpation. If complete resection had not been achieved, or the cut was not judged to be long enough, a second or third cut to the side of midline was performed. The larynx was then sprayed with 21 mL topical anti-inflammatory ‘flush’ through the endoscope (10mls glycerine, 1mls dexamethasone (5 mg/mL) and 10mls DMSO). All horses were then endotracheally intubated and moved to a padded recovery room where supplemental oxygen was administered until spontaneous swallowing was observed to confirm complete function of the larynx. At this time the endotracheal tube was removed and the horse sedated (xylazine 0.75 mg/kg IV) to facilitate a smooth anaesthetic recovery.

Horses underwent post operative endoscopy at 12–24 h post-surgery and the findings were recorded. Post operative treatment with corticosteroids, anti-inflammatory throat flush, NSAIDs or antibiotics was recorded.

### 2.5 Case–control study design, population, data collection and variables

Secondary to the cohort study, a case–control study was conducted. Case horses were horses meeting the case definition in the cohort study. Controls were the three oldest dam-line siblings to the case horse, when three siblings were available. For each horse, the number of career races, the number of races pre- and post-surgery, the total earnings ($AUD) and earning pre- and post-surgery were obtained from Harness Racing Australia (see text footnote 1) website for STBs and from www.racing.com for TBs. Data collection ended in March 2023. For horses that were born after 2020, racing performance was not considered, as it was unlikely to be representative of racing performance.

### 2.6 Outcome variables

**Figure 3:**
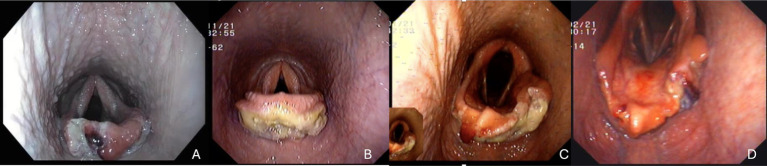
Immediate post operative images (A) mild inflammation with mild epiglottic disfigurement, (B) moderate inflammation with mild epiglottic disfigurement, (C) moderate inflammation with severe epiglottic disfigurement (D) moderate epiglottic disfigurement with mild inflammation.

A short-term complication was defined as any anaesthetic or iatrogenic complication occurring within 48 h of surgery. Upper airway pathology was defined as any surgical complications relating to the upper airway or concurrent airway abnormality identified post-surgery. This included re-entrapment of the epiglottis, epiglottic disfigurement/hypoplasia, dorsal displacement of the soft palate (DDSP) - either permanent or intermittent -, persistent respiratory noise on resuming exercise or any other dynamic airway collapse as identified on dynamic respiratory endoscopy. If no long-term medical records were available, owners were called to obtain information. Epiglottic disfigurement/hypoplasia was graded as mild, moderate or severe. Mild included mild changes to the contour, e.g., thickening of the tip, moderate included mild hypoplasia or more severe changes to the shape/ contour and severe was grossly disfigured or very small. Inflammation was examined as a separate variable and was graded as none (0), mild (1), moderate (2) or severe (3) (Figure 3).

### 2.7 Statistical analyses

Categorical data were described as numbers and percentages. Continuous data were checked for normality and described using medians, interquartile ranges (IQR) and maximums if non-normally distributed and means and standard deviation (SD) if normally distributed.

Associations between the outcome of racing post-surgery (yes or no) and explanatory variables including case signalment, racing performance pre-surgery, and severity score were explored using logistic regression modelling. Three models were built based on the *a priori* determination of the importance of the three post-surgery complications of inflammation post-surgery, airway complications and epiglottic re-entrapment. Univariable analysis was used to screen variables for inclusion in the multivariable model, with variables included in multivariable modelling if the likelihood ratio test (LRT) *p*-value <0.25. Variables were retained in the final model if the LRT *p*-value was ≤0.05. Interactions between variables in the multivariable model were assessed and retained if LRT *p* < 0.05. Associations were presented as odds ratios (OR) and 95% confidence intervals (95% CI) for all three models.

The time to return to racing post-surgery and case signalment, racing pre-surgery, and complications post-surgery were explored using multivariable Cox proportional hazard models. Variables were retained in the multivariable model if the likelihood ratio test (LRT) *p*-value was ≤0.05. The assumption of proportional hazards was assessed using the methods described by Grambsch and Therneau (34), with the assumptions violated if *p* ≤ 0.05 (17).

For matched case–control data, where cases were compared to maternal siblings, conditional logistic analyses were conducted. For each breed, univariable models were generated to identify associations between case status and the outcome of having a race start, with dam included as a conditional effect to account for clustering by dam.

For all analyses, statistically significance was considered as *p* < 0.05. Clinical significance was considered at *p* < 0.10. All statistical analyses were conducted using Stata SE version 18 (StataCorp. 2023. Stata Statistical Software: Release 18. College Station, TX: StataCorp LLC).

## 3 Results

In total there were 82 cases where epiglottic entrapment surgery was performed at least once: 33 STB cases and 49 TB cases. The mean age of horses undergoing surgery was 3.75 (SD 1.77) years, with the mean age of STB cases was 3.99 (SD 1.80) years and the mean age of TB cases was 3.59 (SD 1.75) years. There were 32 (39%) geldings (15 STB and 17 TB), 43 (52%) mares (15 STB and 28 TB), and 7 (9%) colts (3 STB and 4 TB).

The majority of surgeries were conducted by one surgeon (54, 66%). Two surgeons conducted 11 (13%) and 10 (12%) surgeries. Three surgeons performed EE surgeries on STB horses. One surgeon undertook the majority of STB surgeries (24; 73%). Eight surgeons undertook EE surgeries on TB horses and one surgeon undertook the majority of TB surgeries (30; 61%).

### 3.1 Performance pre-surgery

Twelve (15%) cases were less than 2 years of age at the time of surgery, 9 of these were TBs. There was no significant difference between breed and surgery being undertaken on a horse less than 2 years old (*p* = 0.34).

One horse was missing the number of pre-surgical race starts, leaving 69 cases that could have raced prior to surgery. The majority of cases aged 2 or older had raced (*n* = 61/69; 88%). Of the unraced horses, 3 were STB. There was no significant difference between the number that had raced pre-surgery and breed (0.99).

Cases that had raced (*n* = 61), raced a median of 10 (IQR 5 to 31; maximum 124) times. There was a significant difference in the number of pre-surgery starts by breed (*p* = 0.01), TB had a median of 8 (IQR 4 to 15; maximum 55; *n* = 35) and STB had a median of 21 (IQR 7 to 42; maximum 124; *n* = 26). Cases that had raced (*n* = 61), had won a median of $26,100 (IQR 7,180 to $85,850; maximum $727,058) in prizemoney. There was no significant difference in prize money won and breed prior to a case undergoing surgery (*p* = 0.50).

### 3.2 Clinical history

Thirty-five (43%) horses did not have clinical histories available, 27 of these were TBs. Of the horses with clinical histories related to epiglottic entrapment, 13 (16%) had an abnormal finding on a pre-sale endoscopy, 18 (22%) had trainer reported poor performance, 16 (20%) had respiratory noise during exercise, and 6 (7%) had slow recovery post-exercise.

Eight (10%) cases did not have information regarding medical pre-treatment in the clinical notes, 7 of these were TBs. Most cases had pre-treatment prior to surgery (*n* = 52; 63%; *n* = 20; 61% STB and *n* = 34; 58%). If pre-treatment was undertaken, it was conducted for a median of 5 (IQR 5 to 7; maximum 10) days. Medical pre-treatment protocols varied throughout the duration of the study and were dependent on the referring veterinarian and operating surgeon preference. Pre-treatment consisted of a course of non-steroidal anti-inflammatories +/− corticosteroids +/− antibiotics (Typically a combination of phenylbutazone (2.2 mg/kg PO SID) and trimethoprim sulpha (30 mg/kg PO) BID for 5 days).

Three cases (all STBs; 4%) did not have clinical notes regarding pre-surgery endoscopic findings. Of the cases that did, 72 (88%) cases had a severity score, 63 (77%) and 56 (68%) had an assessment of epiglottic membrane ulceration or thickness, respectively (Table 1).

**Table 1:** Pre-surgery endoscopic findings for 30 STBs and 49 TBs undergoing epiglottic entrapment surgery at ABEC between 2011 and 2022.

		Standardbred (*n* = 30)	Thoroughbred (*n* = 49)
Endoscopic finding	Assessment	*n* (%)	*n* (%)
Severity score	Total	25 (83)	47 (96)
	1	15 (60)	16 (34)
	2	8 (32)	10 (21)
	3	0	11 (23)
	4	2 (8)	10 (21)
Epiglottic membrane thickness	Total	14 (47)	42 (86)
	Thin or very thin	12 (86)	21 (50)
	Moderate	1 (7)	0
	Thick	1 (7)	21 (50)
Ulceration	Total	22 (73)	41 (84)
	Ulcer present	10 (46)	28 (68)

The severity score was calculated by adding together the subjective scores for membrane thickness and presence of ulceration on immediate pre-operative resting scope. Entrapping membrane thickness was graded as mild (=1), moderate (=2) or severe (=3) along with the presence of ulceration (ulcerated = 1, non-ulcerated =0) The scores, were added together to calculate an overall severity score.

### 3.3 Post-surgical outcomes

#### 3.3.1 Anaesthesia/surgical complications

Two TB cases experienced a complication during recovery from anaesthesia: one case died in recovery (postmortem examination was unable to identify a definitive cause of death), and one case experienced a minor, superficial distal limb laceration. Three cases experienced a complication in the 48 h post-surgery related to anaesthesia: one case each of pleuropneumonia (TB), inappetence (TB) and rectal impaction (STB). No iatrogenic surgical complications were reported.

#### 3.3.2 Inflammation

The presence of inflammation following surgery was noted in 75 cases. Eleven (15%) cases had no inflammation (five STB), 35 (47%) had mild inflammation (16 STB), 16 (21%) had moderate inflammation (six STB) and 13 (17%) had severe inflammation (four STB).

There were no significant associations between the breed (*p* = 0.83), age (*p* = 0.71), sex (*p* = 0.27), surgeon (*p* = 0.70), pre-treatment (p = 0.83), severity grade (*p* = 0.41) or racing pre-surgery (*p* = 0.82) and whether or not a case experienced inflammation post-surgery.

#### 3.3.3 Airway complications

In total, 17 (21%) of cases had upper airway pathology following surgery; seven STBs and ten TBs. Ten (12%) cases had a deformed or hypoplastic epiglottis (seven TB), five (6%) cases had DDSP (one TB), three (4%) cases made a respiratory noise (one TB), seven (9%) cases required a second surgery due to a re-entrapped epiglottis. Of these, three were STB cases and four were TB cases.

There was a clinically relevant association between the medical pre-treatment of cases and post-surgery airway complications (*p* = 0.10). Of the cases with pre-treatment information, 35% (*n* = 7/20) and 15% (*n* = 8/54) of cases without and with pre-treatment, respectively, had a post-surgery airway complication.

There was an association between the severity grade noted on pre-surgery endoscopy and post-surgery airway complications (*p* = 0.07). Overall, 6 (50%) cases with a grade 3 or above severity score pre-surgery experienced a post-surgery airway complication compared to four (13%) and three (17%) with a grade 2 or 1, respectively, severity score pre-surgery experiencing post-surgery airway complications.

There were no significant associations between the breed (*p* = 0.57), age (*p* = 0.96), sex (*p* = 0.50), surgeon (*p* = 0.29), or racing pre-surgery (*p* = 0.51) and whether or not a case experienced upper airway pathology post-surgery.

There were no significant associations between the breed (*p* = 0.99), age (*p* = 0.41), sex (*p* = 0.84), surgeon (*p* = 0.19), pre-treatment (*p* = 0.20), severity score (*p* = 0.46) or racing pre-surgery (*p* = 0.99) and whether or not a case experienced a re-entrapment post-surgery.

Cases with no airway complications had a median of 2 (IQR 1 to 3; maximum 20) days in hospital. Cases with airway pathology other than re-entrapment had a median of 2 (IQR 1 to 8; maximum 10) days in hospital and those with re-entrapment had a median of 5 (IQR 1 to 6; maximum 9) days in hospital (*n* = 81).

### 3.4 Performance post-surgery

There were 78 cases that did not die (*n* = 1) or retire (*n* = 1) post-surgery due to anaesthetic complications and were more than 2 years of age at the time of surgery in 2022 (*n* = 2) and were eligible for inclusion in the performance post-surgery part of the study. The excluded cases included one STB and three TBs.

In total, 59 (74%) of cases raced following surgery. Based on logistic regression modelling, cases with severe inflammation post-surgery (*p* < 0.001; OR 0.02; 95% CI 0.001 to 0.006) or airway complications (*p* < 0.001; OR 0.09; 95% CI 0.02 to 0.34) or an epiglottic re-entrapment (*p* < 0.01; OR 0.09; 95% CI 0.01 to 0.54) were at a lower odds of racing post-surgery when other variables were accounted from Table 2.

**Table 2:** Logistic regression analysis of associations between whether a case undergoing epiglottic entrapment surgery at ABEC raced post-surgery.

Variable	Level	Raced post-surgery *n* (%)	Total *n* (%)	Odds ratio	95% Confidence interval	*p*-value*
Airway complication (*n*=78)	No	53 (84)	63	Reference		**<0.001**
	Yes	6 (40)	15	0.09	0.02–0.34	<0.001
Sex	Gelding	26 (84)	31	Reference		**0.07**
	Filly or mare	30 (73)	41	0.48	0.13–1.83	0.28
	Colt or stallion	3 (50)	6	0.09	0.01–0.66	0.02
Epiglottic entrapment (*n* = 78)	No	57 (97)	71	Reference		**<0.01**
	Yes	2 (3)	7	0.09	0.01–0.54	0.01
Raced prior to surgery	No	11 (58)	19	Reference		**0.04**
	Yes	48 (81)	59	3.49	1.05–11.59	0.04
Inflammation post-surgery (*n* = 72)	None	9 (82)	11	Reference		**<0.001**
	Mild	27 (82)	33	0.75	0.1–5.66	0.78
	Moderate	12 (80)	15	0.49	0.05–4.98	0.55
	Severe	5 (38)	13	0.02	0.001–0.3	0.00
Sex	Gelding	23 (82)	28	Reference		**0.02**
	Filly or mare	27 (71)	38	0.13	0.01–1.11	0.06
	Colt or stallion	3 (50)	6	0.03	0.002–0.58	0.02
Raced prior to surgery	No	10 (56)	18	Reference		**0.05**
	Yes	43 (80)	54	4.1	1.009–16.67	0.05

Data included cases undergoing epiglottic entrapment surgery between 2011 and 2022 and could have returned to racing (*n* = 78; 31 Standardbred cases and 46 Thoroughbred cases). *p-values in bold represent the Likelihood ratio *p*-value, remaining *p*-values are Wald *p*-values.

Based on logistic regression modelling, there was a significant association between racing post-surgery and the sex of the case (*p* = 0.02), whether the case had raced pre-surgery (*p* = 0.05) and the level of inflammation reported during endoscopy post-surgery (*p* < 0.001). Cases that had raced prior to surgery were 4.1 times (95% CI 1.01–16.67) more likely to race post-surgery than unraced cases. Cases with severe inflammation post-surgery were at a reduced odds (OR 0.02; 95% CI 0.001 to 0.30) of racing post-surgery compared to cases with no inflammation noted post-surgery.

Cases with no airway complications that returned to racing post-surgery (*n* = 52) had a median of 12 (IQR 5.5 to 28.5; maximum 194) starts post-surgery and won a median of $23,051.50 (IQR $6,585 to $53,022.50; maximum $388,550) in prize money. Cases with airway complications other than re-entrapment and returned to racing (*n* = 5) had a median of 22 (IQR 7 to 22; maximum 27) starts post-surgery and won a median of $8,000 (IQR $7,166 to $26,583; maximum $49,640) in prize money. The two cases with re-entrapment that raced post operatively had 50 and 62 starts post-surgery and won $50,625 and $38,740 in prize money, respectively.

Cases took a median of 123 (IQR 93 to 180; maximum 927; *n* = 58) days to return to racing following surgery. Time to return to racing was significantly associated with age at the time of surgery (*p* < 0.01), breed (*p* < 0.001), sex (*p* < 0.001), whether or not a case had raced pre-surgery (*p* < 0.001) and whether a case had experienced re-entrapment post-surgery (*p* < 0.001). Based on the Cox proportional hazards model, cases with epiglottic re-entrapment took 97% longer to return to racing compared to cases without re-entrapment. TB cases took 79% longer to return to racing compared to STB cases. Geldings returned to racing 65% faster than fillies or mares. Cases with no airway complications took a median of 117 (IQR 91 to 180; maximum 927) days, those with an airway complication but not re-entrapment took a median of 151 (IQR 150 to 160; maximum 211) days. The two cases with re-entrapment took 154 and 455 days (Table 3).

**Table 3:** Survival analysis for time to return to racing for cases that returned to racing following epiglottic entrapment surgery.

Variable	Level	Raced post-surgery *n* (%)	Days to return to racing median (IQR)	Hazard ratio	95% Confidence interval	*p*-value*
Age at surgery (years)	<3	10	497 (160–756)	0.24	0.05–1.18	0.08
	3	12	115.5 (77.5–200)	Reference		**<0.01**
	4	17	126 (117–157)	0.17	0.06–0.47	0.001
	>4	20	105 (88.5–154)	0.32	0.13–0.78	0.01
Breed	Standardbred	24	94 (67–155.5)	Reference		**<0.001**
	Thoroughbred	35	141 (116–246)	0.21	0.09–0.45	<0.001
Raced pre-surgery	No	11	539 (246–756)	Reference		**<0.001**
	Yes	48	117 (88.5–152.5)	17.15	3.77–77.98	<0.001
Sex	Filly or mare	26	116.5 (92–157)	Reference		**<0.001**
	Gelding	30	123 (108–241)	1.65	0.81–3.34	0.17
	Colt or stallion	3	451 (446–742)	0.07	0.01–0.34	0.001
Re-entrapment	No	57	120 (93–180)	Reference		**<0.001**
	Yes	2	304.5 (154–455)	0.03	0.004–0.23	0.001

^*^p-values in bold represent the Likelihood ratio *p*-value, remaining *p*-values are Wald *p*-values.

A comparison between pre-racing performance index and post-surgery racing index can be found in the Supplementary Tables A, B.

### 3.5 Performance compared to match siblings

Of the 77 cases that underwent surgery for epiglottic entrapment and were included in the post-surgery performance part of the study, 76 had at least one maternal sibling available for comparison; seven had one sibling, seven had two siblings and 62 had three matched maternal siblings. Four cases did not have a maternal sibling, two of them did not have an airway complication post-surgery. All four cases without maternal siblings were Standardbreds.

The 76 cases had a total of 207 matched siblings and a total of 283 horses. In total, 69 (*n* = 76; 91%) case horses and 159 (*n* = 207; 77%) control horses had raced.

Within the Standardbreds, 25 (*n* = 29; 86%) case horses and 67 (*n* = 80; 84%) control horses had raced (Table 4). Within the Thoroughbreds, 44 (*n* = 47; 94%) case horses and 92 (*n* = 127; 72%) control horses had raced. In Standardbreds, cases were no more likely to race compared to their non-case siblings when dam was accounted for (*p* = 0.77). Thoroughbred cases were 5.5 times (95% CI 1.61 to 18.73; *p* = 0.01) more likely to race than their maternal siblings.

**Table 4:** Conditional logistic regression analysis of return to racing for case standardbred and thoroughbred racehorses that underwent epiglottic entrapment surgery at ABEC January 2011–July 2022 (*n* = 76) and matched maternal siblings (*n* = 207).

Breed	Variable	Raced	Total	Odds ratio	95% Confidence interval	*p*-value
Standardbred	Control	67 (84)	80	Reference		
	Case	25 (86)	29	1.19	0.36–3.91	0.77
Thoroughbred	Control	92 (72)	127	Reference		
	Case	44 (94)	47	5.49	1.61–18.73	0.01

Standardbred cases raced a median of 52 (IQR 21 to 102; maximum 199) times, and controls raced a median of 36 (IQR 13 to 78; maximum 321) starts. Cases won a median of $48,930 (IQR $21,170 to $132,719; maximum $720,180) and controls won a median of $40,883 (IQR $13,960 to $82,290; maximum $798,759). Thoroughbred cases raced a median of 15 (IQR 9 to 28; maximum 105) times, and controls raced a median of 13 (IQR 5 to 28; maximum 65) starts. Cases won a median of $49,560 (IQR $28,727.50 to $84,717.50; maximum $517,250) and controls won a median of $24,155 (IQR $5,065 to $61,195; maximum $1,332,375).

## 4 Discussion

To the authors’ knowledge, this is the largest study evaluating the risk factors for returning to racing following surgical treatment of EE, and the first considering the outcomes for STB racehorses.

There was no difference in the likelihood of racing between STBs that underwent EE surgery and their maternal siblings. However, TB cases that underwent EE surgery were more likely to race post-surgery compared with their maternal siblings. Overall, 75% of cases in the current study raced post-surgery, which is similar to recent studies (11, 15). Horses that had raced pre-surgery were 4.1 times more likely to race following surgery than their unraced cohort, which is similar to the results reported by Russell and Wainscott. The cause of the lower likelihood of racing for younger or unraced horses to race post-surgery is unclear. It may reflect the presence of a normal functioning larynx in raced horses with the later acquisition of EE as has been previously reported (13, 14). Unraced horses potentially have underlying pathology pre-disposing them to EE, preventing successful racing. Alternatively, owners may make different decisions for raced compared to unraced horses, highlighting that further investigation is needed to elucidate these differences. Younger horses were slower to return to racing, potentially requiring more training before a race start compared to a previously campaigned racehorse (20, 21). In addition, the likelihood of a successful racing start diminishes with increasing age and with interruptions to training regimens (20, 22, 23). Kieffer et al. identified TB females were more likely to present with EE than their male counterparts, a finding not supported in the current study (5, 6, 13, 14). In the current study, geldings were more likely to race and returned to racing more quickly post-surgery compared to fillies and colts, which could reflect a bias of owners to protect breeding stock. TBs were significantly slower to go back to racing than STBs. This is a novel finding, and the authors hypothesise this may reflect breed differences between the respiratory and physiological demands of the two breeds (18, 19).

In this study, most cases (75/82) had post operative inflammation, and a post-surgical airway complication was observed in 21% of cases. Whether a horse experienced a post-surgical airway complication was related to medical pre-treatment prior to surgery emphasising the importance of medical pre-treatment of EE. The severity score was developed to grade the overall inflammation pre operatively however it requires further validation which is a limitation of its application in this study and could explain the lack of association between the severity score and post operative inflammation. Given the negative impact of inflammation post operatively, future studies could consider the use of objective inflammatory markers (e.g., SAA) to grade and monitor inflammation pre and post operatively (24). This could potentially help to provide prognostic information to better predict cases at a higher risk of post operative complications.

Complications involving the upper airway following EE surgery have not been previously reported. In our opinion, the complications identified were not the result of surgery but most likely related to the underlying disease process. These horses were at reduced odds of racing following surgery. Most horses with suspected upper airway pathology (i.e., a respiratory noise) following surgery did not undergo dynamic endoscopy which would have assisted in characterising the noise (25, 26). Intermittent DDSP following EE surgery is likely underestimated in the current study as this can only be conclusively diagnosed using exercising endoscopy (1, 26, 27). Deformed or hypoplastic epiglottises were common (10/17), and whether this is congenital, the underlying aetiology of the entrapment or a consequence of prolonged entrapment is unclear. Radiography reliably identifies epiglottic hypoplasia (28) and should be considered in chronic, inflamed EE to provide additional prognostic information.

Post-surgery airway complications negatively impact future racing performance and were less likely in horses receiving medical pre-treatment, despite the lack of a standardised pre-treatment protocol. The variation in pre-treatment protocol is limitation due to the retrospective nature of the study. Although another potential source of bias, recommendations from the operating surgeon to pre-treat were not generally based on the initial appearance of the EE on the diagnostic scope but rather surgeon experience and preference- especially with cases that were referred to the hospital which were treated prior to performing laryngeal scoring. This would help to reduce, but not remove potential selection bias that could also potentially have impacted on our findings. All pre operative severity scores used in the analysis were from the appearance of the EE immediately prior to surgery (i.e. after pre-treatment if this occurred).

The author uses 2.2 mg/kg phenylbutazone PO SID and 30 mg/kg trimethoprim sulphonamide PO BID for 5 days and 0.1 mg/kg dexamethasone IV 24 h prior to surgery. Whilst antibiotic therapy has been justified by the assumption of a bacterial component to the inflammation resulting from ulceration of the entrapping membrane, this should be critically reviewed especially considering the importance of antimicrobial stewardship and perhaps abandoned if there is no evidence of ulceration (29). Future prospective studies with a controlled pre-medication protocol would be useful to provide evidence based recommendations to clinicians.

Nine percent of cases in the current study had re-entrapment post-surgery which is similar to previous studies using a similar technique (30–32). These cases were less likely and took longer to return to racing. The reasons for re-entrapment are varied and could be associated with excessive post operative inflammation, may reflect an underlying disease process (hypoplastic epiglottis or excessive aryepiglottic folds) or technique error (e.g., incomplete transection of the entrapping membrane). The described surgical technique is effective with no iatrogenic surgical trauma to the larynx or pharynx reported which is in line with earlier studies using a similar technique and improved compared to other reported techniques (6, 8, 11, 12). The technique is straightforward and whilst all the cases in this study were performed in a hospital, it can be performed in the field, increasing convenience, and reducing costs to the client (14). There were however two severe anaesthetic related complications, (one died and one retired as a broodmare after recovering from severe pleuropneumonia). Equine anaesthesia is associated with a higher risk of mortality than other animals however the anaesthetic for this technique would typically be considered to be low risk (intravenous, fast and elective procedure) (33). Whilst a definitive cause of death was not established for the case that died, we were concerned regarding potential airway obstruction by the surgeon’s hand whilst manipulating the epiglottis during surgery. After this incident, all horses had a nasopharngeal tube placed immediately after induction of anaesthesia for supplementation with 100% oxygen at a rate of 5 L/h.

Although standing techniques eliminate the risk of general anaesthesia, severe, iatrogenic, potentially career ending surgical complications including soft palate transection, severe nasal passage haemorrhage and epiglottis transection/thermal injury are reported (8, 30–32). These complications are avoided with our technique. Of note, all but one of the surgeries in the current study was performed by female surgeons with small glove sizes of 6 and 6.5. Oral access to the pharynx can be challenging with bigger hands (14). Whilst there was no difference in outcome between surgeons, a single surgeon performed 66% of the surgeries in this case series which has the potential to introduce bias. A larger study with more operating surgeons would be helpful to remove the potential for this bias and further examine if there is an influence of the surgeon on the outcome of EE surgery. Clinician experience, glove size, temperament of the horse, client budget and equipment available should be factored when deciding on surgical technique.

One of the strengths, but also a limitation of the current study was the selection of maternal siblings as control horses. This enabled racing performance comparison between intrinsically similar groups of horses. However, it was assumed that matched siblings did not have EE or any other airway or performance limiting abnormality. A previous study found no significant differences between TB cases with EE and maternal siblings (11). This was the expectation in the current study, instead TB cases were 5.5 times more likely to race than their maternal siblings. It may be that the sample size in the current study is larger than presented by Curtis et al. Alternatively, the study population in the previous study was yearlings, whereas in the current study most horses were of racing age and/or had raced prior to surgery. No difference in racing performance was identified between STB cases and control STBS, indicating that STB cases performed as expected when compared to horses without EE. Whilst there is no research into the potential heritability of EE, it is possible that differing genetics pools of horses from different countries may influence the development of EE which could also account for the difference between our study and Curtis et al.

In conclusion, STBs are as likely, and TBs are more likely to race as their maternal siblings following EE surgery however STB return to racing significantly faster than TBs. Having inflammation post-surgery, an airway complication and/or epiglottic re-entrapment reduced the likelihood of a case returning to racing. Medical pre-treatment reduces post operative inflammation and is associated with a lower percentage of post-surgery airway complications. Further efforts to refine and standardise this protocol would be useful to provide recommendations to clinicians performing surgery to correct EE. Older horses, geldings, and cases that had raced prior to surgery were more likely to return to racing.

## Data Availability

The raw data supporting the conclusions of this article will be made available by the authors, without undue reservation.

## Footnotes

^1^ www.hra.com.au/standardbreedbreedregister.com.au
