# Supplementary material for: Comparative analysis of racing performance following surgical correction of epiglottic entrapment in standardbreds and thoroughbreds
Source: Front Vet Sci. 2024 Nov 19;11:1479144. doi: 10.3389/fvets.2024.1479144 (PMC11611841; doi:10.3389/fvets.2024.1479144)
Supplement: Supplementary file 1 [file Table_1.DOCX]

Supplementary tables

The racing performance – number of starts and prize money won – for cases is presented in Supplementary Table A for Standardbreds and Supplementary Table B for Thoroughbreds.

There were no significant differences between complication category and the median starts or prizemoney pre-surgery for Standardbred cases (Supplementary Table A). There were no significant differences between complication category and the median starts or prizemoney post-surgery for Standardbred cases. There was no difference in the median prize money (P=0.23) or median number of starts (P=0.47) pre- or post-surgery for Standardbred cases.

There were no significant differences between complication category and the median starts or prizemoney pre-surgery for Thoroughbred cases (Supplementary Table B). There were no significant differences between complication category and the median starts or prizemoney post-surgery for Thoroughbred cases. There was a significant difference in the median prize money (P=0.03) pre- and post-surgery, with horses with an airway complication earning less in prize money. There was no statistically significant difference between the number of starts pre- and post-surgery for Thoroughbred cases (P=0.08).

It should be noted that the follow up period post-surgery varied for each case, so the post-surgery performance in terms of prize money and number of starts does not necessary indicate the career potential of the case.

Supplementary Table A: Pre- and post-surgery racing performance (number of starts and prizemoney won) for Standardbred horses (n=30) undergoing epiglottic entrapment surgery at ABEC between 2011 and 2022, stratified by post-surgical complication category.

| Timepoint | **Variable** | **Outcome category** | | **Standardbred** | | | | **P-value*** | **P-value**** |
| --- | --- | --- | --- | --- | --- | --- | --- | --- | --- |
|  |  |  | | **Number** | | **Median** | **Interquartile range; maximum** |  |  |
| Pre-surgery (n=26) | Prize money ($AUD) | No complication | | 23 | 26947 | | 3000 - 126956; 727058 | 0.76 | 0.23 |
|  |  | Airway complication | | 1 | 60324 | | 60324 - 60324; 60324 | | |
|  |  | Re-entrapment | | 2 | 14285 | | 9237 - 19333; 19333 | | |
|  |  |  |  |  |  | |  |  |  |
|  | Number of starts | No complication | | 23 | 21 | | 7 - 42; 124 | 0.42 | 0.47 |
|  |  | Airway complication | | 1 | 44 | | 44 - 44; 44 | |  |
|  |  | Re-entrapment | | 2 | 11 | | 7 - 15; 15 | |  |
|  |  |  |  |  |  | |  |  |  |
| Post-surgery (n=23) | Prize money ($AUD) | No complication | | 19 | 12815 | | 4670 - 41174; 130292 | 0.36 |  |
|  |  | Airway complication | | 2 | 16874.5 | | 7166 - 26583; 26583 | | |
|  |  | Re-entrapment | | 2 | 44682.5 | | 38740 - 50625; 50625 | | |
|  |  |  |  |  |  | |  |  |  |
|  | Number of starts | No complication | | 19 | 17 | | 7 - 56; 194 | 0.52 |  |
|  |  | Airway complication | | 2 | 24.5 | | 22 - 27; 27 | |  |
|  |  | Re-entrapment | | 2 | 56 | | 50 - 62; 62 | |  |

* P value indicates a non-parametric Kruskal-Wallis test for within group differences in median starts or earnings

** P-value indicated a non-parametric Kruskal-Wallis test for between pre- and post-surgery differences in median starts or earnings

Supplementary Table B: Pre- and post-surgery racing performance (number of starts and prizemoney won) for Thoroughbred horses (n=49) undergoing epiglottic entrapment surgery at ABEC between 2011 and 2022, stratified by post-surgical complication category.

| Timepoint | **Variable** | **Outcome category** | | **Standardbred** | | | | **P-value*** | **P-value**** |
| --- | --- | --- | --- | --- | --- | --- | --- | --- | --- |
|  |  |  | | **Number** | | **Median** | **Interquartile range; maximum** |  |  |
| Pre-surgery (n=35) | Prize money ($AUD) | No complication | | 26 | 17740 | | 6435 - 51825; 506750 | 0.35 | 0.03 |
|  |  | Airway complication | | 6 | 40067.5 | | 31000 - 166520; 346210 | | |
|  |  | Re-entrapment | | 3 | 85850 | | 2961 - 199465; 199465 | | |
|  |  |  |  |  |  | |  |  |  |
|  | Number of starts | No complication | | 26 | 7 | | 3 - 13; 52 | 0.43 | 0.08 |
|  |  | Airway complication | | 6 | 11 | | 6 - 31; 55 | |  |
|  |  | Re-entrapment | | 3 | 12 | | 4 - 18; 18 | |  |
|  |  |  |  |  |  | |  |  |  |
| Post-surgery (n=36) | Prize money ($AUD) | No complication | | 33 | 30500 | | 10500 - 57425; 388550 | 0.41 |  |
|  |  | Airway complication | | 3 | 8000 | | 4730 - 49640; 49640 | | |
|  |  | Re-entrapment | |  |  | |  | | |
|  |  |  |  |  |  | |  |  |  |
|  | Number of starts | No complication | | 33 | 11 | | 5 - 21; 92 | 0.59 |  |
|  |  | Airway complication | | 3 | 7 | | 3 - 22; 22 | |  |
|  |  | Re-entrapment | |  |  | |  | |  |

* P value indicates a non-parametric Kruskal-Wallis test for within group differences in median starts or earnings

** P-value indicated a non-parametric Kruskal-Wallis test for between pre- and post-surgery differences in median starts or earnings
